# Implementation of Basic Life Support training in schools: a randomised controlled trial evaluating self-regulated learning as alternative training concept

**DOI:** 10.1186/s12889-020-8161-7

**Published:** 2020-01-13

**Authors:** Christoph Süss-Havemann, Janina Kosan, Thomas Seibold, Nils Martin Dibbern, Anne Daubmann, Jens Christian Kubitz, Stefanie Beck

**Affiliations:** 1 Department of Anaesthesiology, University Medical Center Hamburg-Eppendorf, Martini-Str. 52, 20246 Hamburg, Germany; 2 Department of Anaesthesiology, Intensiv Care and Pain Management, Kath. Marienkrankenhaus, Alfredstraße 9, 22087 Hamburg, Germany; 3 Specialist Center for Anaesthesia and Pain Medicine, Schoen Clinic Hamburg Eilbek, Dehnhaide 120, 22081 Hamburg, Germany; 4 Department of Medical Biometry and Epidemiology, University Medical Center Hamburg-Eppendorf, Martini-Str. 52, 20246 Hamburg, Germany

**Keywords:** Basic life support training, Self-regulated learning, Self-efficacy, School children

## Abstract

**Background:**

The Kids save lives statement recommends annual Basic Life Support (BLS) training for school children but the implementation is challenging. Trainings should be easy to realise and every BLS training should be as effective as possible to prepare learners for lifesaving actions. Preparedness implies skills and positive beliefs in the own capability (high self-efficacy).

**Methods:**

This randomized controlled cluster study investigates, if self-regulated learning promotes self-efficacy and long-term retention of practical BLS skills. Students in the age of 12 years participated in a practical training in BLS and a scenario testing of skills. In the control group the practical training was instructor-led. In the intervention group the students self-regulated their learning processes and feedback was provided by the peer-group. The primary outcome self-efficacy for helping in cardiac arrest after the training and 9 months later was analysed using a multilevel mixed model. Means and pass-rates for BLS skills were secondary outcomes.

**Results:**

Contrary to the assumptions, this study could not measure a higher self-efficacy for helping in cardiac arrest of the students participating in the intervention (*n* = 307 students) compared to the control group (*n* = 293 students) after training and at the follow-up (mean difference: 0.11 points, 95% CI: − 0.26 to 0.04, *P* = 0.135). The odds to pass all items of the BLS exam was not significantly different between the groups (OR 1.11, 95% CI: 0.81 to 1.52, *p* = 0.533). Self-regulated learning was associated with a higher performance of male students in the BLS exam (mean score: 7.35) compared to females of the intervention (female: 7.05) and compared to males of the control (7.06).

**Conclusion:**

This study could not resolve the question, if self-regulated learning in peer-groups improves self-efficacy for helping in cardiac arrest. Self-regulated learning is an effective alternative to instructor-led training in BLS skills training and may be feasible to realise for lay-persons. For male students self-regulated learning seems to be beneficial to support long-term retention of skills.

**Trial registration:**

ISRCTN17334920, retrospectively registered 07.03.2019.

## Background

Ischemic heart disease is one of the leading causes of death in the world. According to international consensus, the most important determinant to survive a sudden cardiac arrest is the presence of a trained rescuer who is ready, willing, and able to act [1, 2]. The Introduction of CPR training for school children is an effective intervention to improve bystander-CPR-rates and increased survival of out-of-hospital cardiac arrest two- to four-fold [3].

Based on the existing evidence the joint-statement “Kids save lives” was released. The statement aims to introduce annual CPR training in the curriculum for all school children world-wide and was endorsed by the World Health Organization in January 2015 [4].

Teachers, physicians, nurses, students and emergency medical staff engage in the training of school children, but the implementation is difficult. The evaluation of nationwide programs demonstrate that not all students were reached and some have been trained only once in their schooldays [5–8]. Important barriers of implementation in schools are lack of instructors and equipment and assumed high costs [7]. In turn there are some factors associated with good implementation rates. These factors are awareness of mandating legislation and successful implementation at other schools. As well as a person in charge for the implementation and teachers, who feel competent to conduct trainings [5].

There is a need for training concepts, which are very effective and focus on the factors strongly associated with patient outcome on the one hand and simple and easy to realise at the other hand.

Training should focus on skills, because effective CPR is crucial for survival. But skills are not the only constitutional factor of CPR by bystanders [1]. The potential rescuer needs enough confidence e.g. perceived ability to perform effective CPR as well. Training improves the belief of potential-rescuers that they can perform CPR effectively. But even trained people only perform CPR in 30–55% of the given occasions [9, 10]. The main reasons for denying live saving cardiopulmonary resuscitation to a patient are fear and lack of confidence of potential-rescuers [11–13].

To overcome fear and lack of confidence training methods should focus on positive beliefs and capabilities to master prospective situations. For conceptual design and measurement of effective teaching, self-efficacy (SE - a person’s belief in his/her capability to organise and execute the course of action required to produce given attainments [14] based on the concept Bandura) is a promising target. Schrunk demonstrated that children derive more SE by observing others, who are similar to themselves (peers) succeeding at a task in contrast to observing adults and showed that training concepts which foster self-regulation processes improve SE [15, 16]. 

Self-regulation processes during learning are not only helpful to stimulate the perceived ability to perform a task but as well for the retention of skills [17]. The literature discriminates between the learning processes relevant for skill acquisition and retention. Immediate feedback of a teacher improves in many contexts learning tempo and higher initial performance. Self-regulation processes like setting learning goals, monitoring the own performance and adapting strategies to achieve these learning goals is positively associated with skill retention [18, 19]. Therefore this study hypothesised that self-regulated practical learning will increase the SE of school children for helping in cardiac arrest immediately after the training and until follow up 9 months later and will have a positive effect on the retention of practical BLS skills.

## Methods

A randomized, rater blinded, controlled cluster study was performed to evaluate the effect of two different training methods. The trial was conducted in September 2016 and the follow up was performed 9 months later in June 2017.

### Participants

The participating schools were recruited from the pool of cooperating high-schools of the Department of Anaesthesiology of the University Hospital Hamburg-Eppendorf. The students of grades seven and eight were randomised class-wise into the intervention- and control arm in a 1:1 allocation ratio. The randomisation was performed in advance by drawing balls of two colours blindly. A cluster was represented by students of one class. All students and their legal guardians were informed in advance about the training and the study goal. Only assessments of students who had their written informed consent to participate on hand were included in the analyses.

### Instruments

Practical skills were evaluated during a 3 min scenario testing using a nine point standardised checklist. The assessment has been developed for school settings and high inter-rater reliability has been shown in a previous study [20]. Demographic data and the self-efficacy were assessed before the practical assessment using a questionnaire. The self-efficacy (SE) was measured with a four-point Likert scale in three dimensions with two questions for each by adding the values of the two questions. The dimensions were helping in general, helping in cardiac arrest and diminished emotional arousal to cardiac arrest. The questionnaire, based on the general self-efficacy scale of Schwarzer and Jerusalem [21] was transformed into a special self-efficacy scale following the authors recommendations (Table 1). The questionnaire was piloted and demonstrated higher SE of students, who were promoted to be a BLS instructor compared to students just participating in a BLS training [22]. Demographic data included age, gender, weight, height and previous CPR training.

**Table 1:** Items of the self-efficacy questionnaire

Domain: helping in general	
1. I can help other people if I try hard.	
2. When I want to help other people, I am certain that I can accomplish my goals.	
Domain: helping in cardiac arrest	
3. In a cardiac arrest situation I am confident that I could deal efficiently.	
4. I can handle the situation if cardiac arrest comes my way.	
Domain: diminished emotional arousal to cardiac arrest	
5. Thanks to my resourcefulness, I can handle unforeseen situations in a resuscitation-situation.	
6. I can remain calm when facing a cardiac arrest situation because I can rely on my coping abilities.	
Response format: 1 = Not at all true 2 = Hardly true 3 = Moderately true 4 = Exactly true	

### Procedure

Training and initial assessment of the students were part of a CPR training event at the participating schools. The CPR training event covered three school lessons (45 min each) and consisted of three parts. Part one was a 45 min interactive lecture on basic life support and AED use. Part two was 45 min of practical training on BLS and AED skills performed in small groups (16–24 students with two trainers). Part three was the assessment.

The practical training during part two was different between the intervention and control group. In both groups, the practical training was performed by trained medical students or high school students (both in their final year before graduating) in the class rooms of the students and based on the four step-approach established by Peyton. The four steps include 1. demonstration, 2. deconstruction, 3. comprehension and 4. execution of the learned. In the control group the skills were trained following the four step-approach. In the intervention the trainers performed step one and two (demonstration and deconstruction). During step three and four the students guided their learning self-regulated and the trainer supervised and supported the communication process. The students were split into small groups of 8 to 12 students and in turns the children took over the part of the instructor, the executor or the rater of BLS. One student explained how and what to do next, two students performed BLS and the other students evaluated the performance and gave feedback afterwards. To support the evaluating and feedback-process the children used training cards with all relevant BLS items. The trainers stimulated the children to guide their feedback based on the performance parameters on the training cards.

The assessment of the practical BLS skills during part three was structured as an OSCE using MiniAnne-mannequins® (LaerdalTM). The medical students/ high schools students were randomly assigned to an assessment station. The students themselves were randomly assigned to the assessment stations before entering the gym. For assessment, the raters used a structured rating checklist with nine binary items. To pass the practical assessment in total, all nine points had to be rated with yes. All raters were trained in advance to use the structured rating-checklist.

At the initial assessment directly after the training the probability of a rater knowing about the group affiliation differed from 1:6 to 1:2. The follow-up assessment was identical. But medical students evaluated the students and were totally blinded of group affiliation.

All trainers were invited to a seminar prior to the training in schools which included five parts [23]. The trainers were randomised between part four and five to intervention or control group trainers. The randomization was performed by letting the trainers choose a specific class without knowing if the class was randomised to the intervention or control group. In the fourth part the two groups were separated, informed about their training concept and practiced it in a simulated BLS teaching session.

### Data analysis

The primary outcome was the self-efficacy for helping in cardiac arrest immediately after the training and at the follow-up 9 months later. Secondary outcomes were pass-rates and means for BLS performance immediately and 9 months later.

#### Sample size calculation

Sample size calculation was based on an estimated difference between the self-efficacy for helping in cardiac arrest of 0.25 points (SD ± 1) between the intervention and control. We assumed, that the SE is higher in the intervention group. With an α of 0.05 (two-sided) and a power of 0.8, fifteen classes with 22 students per group had to be analysed assuming an intracluster correlation of 0.01. We decided to include as many classes as possible.

#### Statistics

The rating sheets were machine readable, electronically scanned and imported into Microsoft Excel. After checking for plausibility, the data was analysed together with a statistician of the Department of Medical Biometry and Epidemiology of the Medical University of Hamburg using SPSS, version 24 (IBM Corp, Armonk, NY, USA).

Descriptive statistics were evaluated for all randomized students by group. For the categorical variables, the absolute and relative frequencies were calculated. Means and standard deviation (SD) were determined for continuous variables.

Assuming a dependence of SE on gender and the intervention and a dependence of practical skills on time and the intervention, we considered gender and its interactions with group and time additionally in our model. Therefore primary and secondary continuous outcomes were analysed using a multilevel mixed model with group, gender and time as fixed effects, class as random effect and time as a repeated effect. In the initial model the three-way- and all two-way-interactions between the three variables were included. Afterwards a stepwise backward elimination of the not significant interactions was conducted. For the binary outcome (passing OSCE), a mixed logistic regression was evaluated with the same specifications as in the model for continuous outcomes. Mean differences and odds ratios with the corresponding 95% confidence intervals (CI) were reported, respectively. Intra-Cluster-Correlations (ICC) were also presented. Two-sided *p*-values < 0.05 were considered as significant.

## Results

**Fig. 1:**
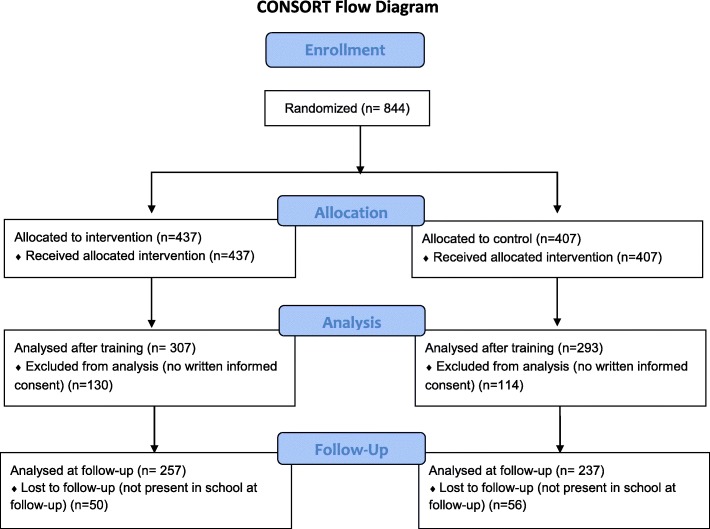
Flow-chart of participants according to CONSORT recommendation

847 students were randomized and trained. 247 did not have written informed consent and the data of these students was not included in the analyses. Data of 307 students of the intervention group (20 Clusters) and 293 students of the control group (21 Clusters) could be analysed immediately after the training. 257 of the intervention group students and 237 of the control group students participated at the follow up nine months later (Fig. 1). Demographic data were comparable between the groups and presented in Table 2. The proportion of the variance, attributable to the students, was higher than the proportion of the variance attributable to the classes for all continuous outcomes (Table 3).

**Table 2:** Demographic data of the students

	Intervention (*n* = 307)	Control (*n* = 293)
Age–yr (S.D.)	12 (1)	12 (1)
Male gender – no. (%)	139 (45.3)	147 (50.2)
Height–cm (S.D.)	160 (7.8)	161 (8.6)
Weight– kg (S.D.)	46.5 (8.0)	47.6 (9.4)
CPR-training < 1 year – no. (%)	4 (1.4)	5 (1.7)
CPR-training > 1 year – no. (%)	16 (5.2)	13 (4.4)
No CPR-training ever – no. (%)	284 (92.5)	274 (93.5)

**Table 3:** Intra-Cluster-Correlations (ICC) for continuous outcomes

Outcome	ICC of the classes	ICC of the students
SE helping in general (% of total variance)	3.38	20.98
SE helping in cardiac arrest (% of total variance)	0.64	17.03
SE diminished emotional arousal (% of total variance)	2.09	15.82
Number of passed items in BLS (% of total variance)	6.38	7.33

For the main outcome of self-efficacy for helping in cardiac arrests no significant difference between the groups (mean difference for helping in cardiac arrest: 0.11 points, 95% CI: − 0.26 to 0.04, *P* = 0.135) was found. Mean scores of male students of the control were descriptively higher compared to male students of the intervention and female students of both groups (Table 4). SE for helping in cardiac arrest decreased significantly over time (mean difference: 1.08 points, 95% CI: 0.97 to 1.20, *p* < 0.001).

**Table 4:** Means of self-efficacy and means of passed items of the BLS exam

		Intervention		Control	
		after training	follow-up	after training	follow-up
SE helping in general (mean; SD)	all	6.88 (1.03)	6.37 (0.95)	7.03 (0.96)	6.47 (1.04)
	male	6.81 (1.08)	6.29 (0.99)	7.01 (0.95)	6.48 (1.07)
	female	6.93 (1.00)	6.44 (0.90)	7.06 (0.97)	6.45 (1.01)
SE helping in cardiac arrest (mean; SD)	all	7.09 (1.02)	6.02 (1.10)	7.20 (0.90)	6.13 (1.21)
	male	7.10 (1.08)	6.02 (1.24)	7.38 (0.85)	6.19 (1.32)
	female	7.09 (0.97)	6.02 (0 .98)	7.03 (0.92)	6.07 (1.07)
SE diminished emotional arousal (mean; SD)	all	6.05 (1.17)	5.09 (1.33)	6.23 (1.13)	5.27 (1.23)
	male	6.05 (1.20)	5.11 (1.39)	6.30 (1.22)	5.29 (1.25)
	female	6.04 (1.15)	5.08 (1.28)	6.16 (1.03)	5.25 (1.22)
Number of passed items in BLS (mean; SD)					
	male	7.66 (1.21)	7.05 (1.54)	7.37 (1.43)	6.72 (1.49)
	female	7.61 (1.13)	6.49 (1.58)	7.41 (1.45)	6.77 (1.48)

For helping in general and diminished emotional arousal to cardiac arrest the control group had significant higher means (mean difference for helping in general: 0.18 points, 95% CI: 0.01 to 0.35, *P* = 0.038), mean difference for diminished emotional arousal: 0.20 points, 95% CI: 0.01 to 0.39, *P* = 0.045).

A significant decrease over time was observed *P* < 0.001 (helping in general: 0.54 points, 95% CI: 0.44 to 0.65; diminished emotional arousal: 0.96 points, 95% CI: 0.82 to 1.09).

Observed means separated for group, gender and time are presented in Table 4.

A mean pass-rate of 25% of all students without a significant difference between the groups was detected (OR 1.11, 95% CI: 0.81 to 1.52, *p* = 0.533). The odds to pass the exam declined significantly over time without any interaction between groups. The chance to pass the exam was 2.64-fold higher directly after the training than nine months later. (OR 2.64, 95% CI: 1.88 to 3.69, *P* < 0.001). Gender had no significant influence on the odds to pass.

For number of passed items in BLS, the interaction between group and gender was not significant with *p*-value of 0.052. The significance was just missed. In this case, nevertheless, we kept the interaction in the model. Male students of the intervention had passed more items than the female in the intervention (male: 7.35, 95% CI: 7.11 to 7.58, female: 7.05, 95% CI: 6.82 to 7.27, difference: 0.30, 95% CI: 0.06 to 0.55, *P* = 0.015). In the control group, no significant difference between male and female students was found. For female and male students, the scores did not differ significantly between the groups (female: intervention: 7.05, 95% CI: 6.82 to 7.27; control: 7.10, 95% CI: 6.86 to 7.33, difference: -0.05, 95% CI: − 0.37 to 0.26, *P* = 0.746; male: intervention: 7.35, 95% CI: 7.11 to 7.58; control: 7.06, 95% CI: 6.83 to 7.29, difference: 0.29, 95% CI: 0.03 to 0.61, *P* = 0.073). Time had a significant influence on number of passed items (mean difference: 0.76, 95% CI: 0.60 to 0.92, *P* < 0.001). Observed means separated for group, gender and time are presented in Table 4.

**Fig. 2:**
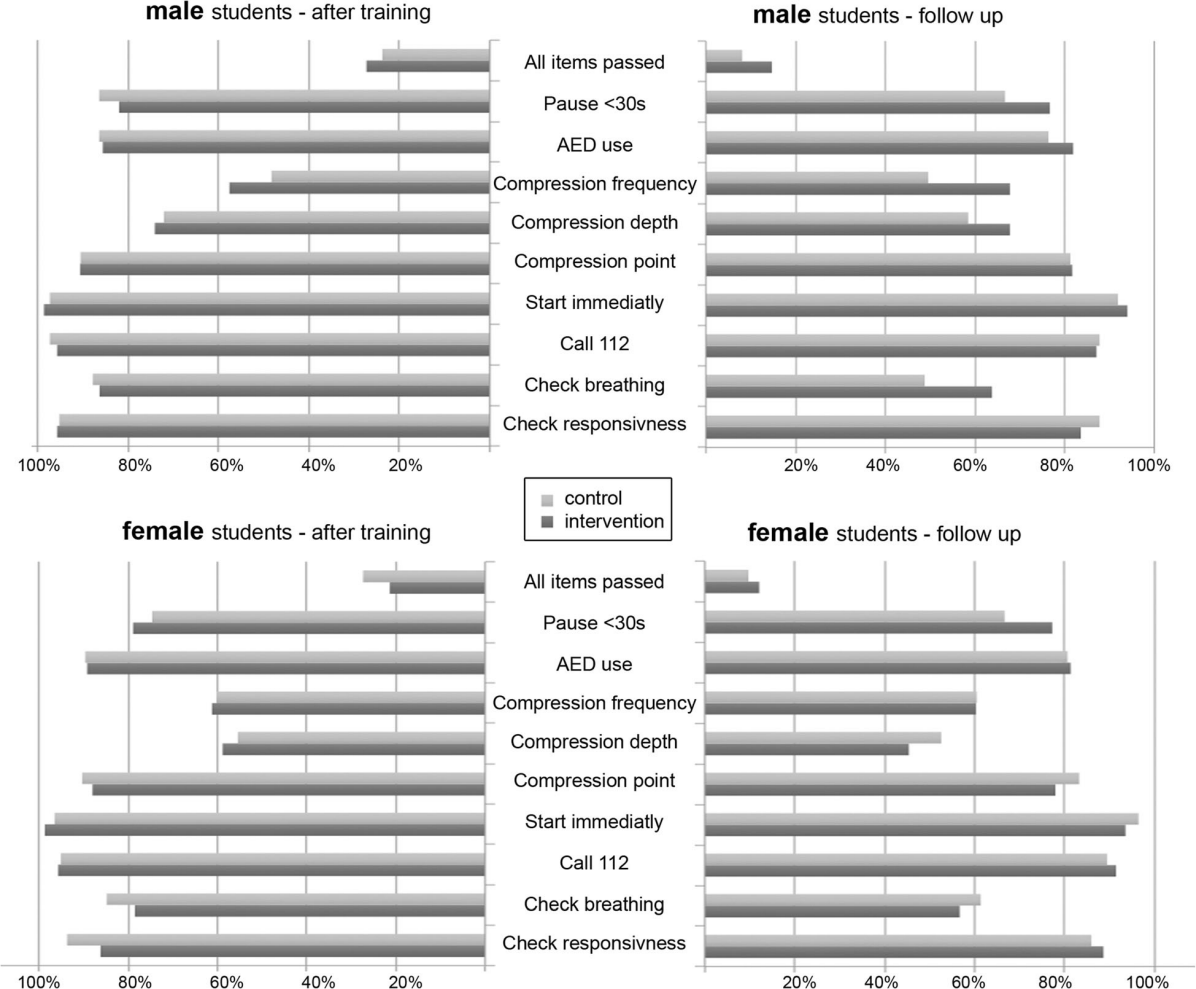
Pass-rates for the BLS-exam and on item level. In the panels at the top the results of the male and at the bottom the results of the female participants are presented. Panels on the left show results immediately after training and on the right nine months later

Descriptive analyses (Fig. 2) showed that male students of the intervention had higher pass-rates for correct compression frequency and depth after the training and at the follow-up compared to females of the intervention. Males of the intervention had better results for assessing breathing, compression frequency, compression depth and pauses less than 30 s compared to male students after control at the follow-up. Male students of the intervention performed chest compression after nine months nearly as good as immediately after training. After nine months all groups passed about 0.6 to 0.7 items less; except the males in the control group declined about 1.1 items.

Independent of the group, the pass rates for checking breathing declined strongly approximately 20% over time. The decline of pass-rates for the other items ranged within 10%.

## Discussion

This study demonstrates that a high percentage of students had self-confidence to help effectively in cardiac arrest after the training and nine months later. Practical skills of the students were high after both training concepts and male students seem to benefit from self-regulated training. Because the instructors used moderation skills but disclaimed feedback based on their clinical skills in the self-regulated training, even lay people may realise self-regulated learning when using videos for the initial demonstration and deconstruction of BLS skills.

Based on the literature we had expected a positive effect of self-regulated learning on the SE for helping in cardiac arrest, because the intervention is grounded on the theoretical concept to support SE developed of Bandura and included recommended instructional strategies to boost SE in the context of medical learning [24]. Evidence based strategies as “setting of challenging and proximal goals”, “providing honest and explicit feedback”, “use of peer modelling” and “facilitation of accurate calibration of SE” were part of the self-regulated concept but did not improve SE for helping in cardiac arrest. Additionally, a significantly lower SE for helping in general was measured in the intervention compared to the control-group, which served as a control variable, and there were also lower scores for diminished emotional arousal.

In our opinion, a possible explanation is the so called “calibration effect”. Immediate feedback that encourages students to shift their focus from actual performance to performance monitoring and evaluation is a strong promotor of self-efficacy calibration and learning [25, 26]. The featured role of peer feedback in the intervention could have resulted in good self-efficacy calibration and reduction of common overestimation of SE in the male group [27, 28]. This thesis is supported by the fact, that the males of the control had the highest SE scores but the lowest scores for BLS at the follow up. The literature reports some evidence, that female students possess and use more self-regulated learning strategies at this age than males [28]. Therefore, a teaching method that promotes self-regulation may be less effective for females at this age.

The results support the thesis, that self-regulated learning leads to good calibration of SE especially in male students but the study missed to measure the accuracy of SE judgment of the students. Cleary [29] summarized methods that allow measuring over- or underestimation of SE. For our setting it would be feasible to let the students estimate if they can perform certain items correctly or to guess the grade they would achieve in the practical assessment and compare it with the actual performance.

Additionally, we recommend using questionnaires to evaluate SE for helping in cardiac arrest with more discrimination power at the upper end for further studies.

In this study the pass-rates of about 25% in the BLS assessment appear low. There are two possible reasons. The students were very young and the assessment was very restrictive.

Students with a mean age of 14 years and a mean weight of 55 kg, who had participated in 2013/14 in our BLS-training with the same training setting and assessment, had achieved pass-rates of about 40% [30]. Compared to the students with 14 years, the 12-year-old students of this study had about 20% lower pass-rates for the items compression depth, compression rate and pauses less than 30 s. This is in line with the literature demonstrating higher physical capacity to maintain effective chest compression with higher age and higher weight [31].

With respect to retention of practical skills, self-regulated learning is only beneficial for male students. The difference between the males and females in the intervention is mainly attributable to long-term retention of practical skills of chest compression. The males of the intervention scored higher than the males of the control because of better retention for breathing check, chest compression quality and less pauses. Up to date there are only some studies reporting an interaction between gender and training method in medical education, because gender is not routinely considered as variable in the analysis [31]. Sopka et al. demonstrated that the training environment interacts with learning of different genders. Female medical students improved CPR skills only in the female only group and not in mixed-gender groups [32].

### Strengths

The study was a randomised controlled trial with a scenario testing of practical skills and an assessment of SE of students including a mid-time follow-up after two different training methods. This study focused on the efficacy of training methods in a real world setting with trained medical students or high school students as facilitors and students of grades seven and eight as learners. The objectivity and reliability of the of the SE- and practical skills assessment can be estimated high because standardised assessment instruments were used, the assessors were blinded for group allocation. The variance within the results for the SE and number of passed items is mainly attributable to difference between the students and not between the classes. The low variance on class level indicates high reliability of the instrument and a slight influence of the trainer on the results. The sample size was high enough to detect a significant interaction between training method and gender on the mid-term BLS performance.

### Limitations

But the study was powered to evaluate the effectiveness of self-regulated learning on the SE to help in cardiac arrest. We expected higher SE of the students after self-regulated learning. There are two explanations for failing to measure a difference. First, all groups scores above 7 (of an eight-point scale) initially and above 6 at the follow-up were observed for helping in cardiac arrest. The discriminating power between the single subjects and groups was limited due to a ceiling effect of the used SE scale.

Second, we probably observed better calibration of the SE in the male group after the intervention. To assess the accuracy of SE calibration additional measures must be added in future studies on self-efficacy. According to the literature good SE calibration is a strong predictor for future successful performance and effective chest compression is essential in resuscitation. The effect of the intervention on behaviour in a real-world situation can’t be tested.

The results depend on how self-regulated learning was realised in this study. If the self-regulated learning concept brings more teachers to become facilitators of BLS training and helps to support the implementation, can’t be predicted. The teachers do not need formal training in BLS to provide self-regulated learning opportunities but must be familiar with the training material and motivated to create opportunities.

## Conclusions

This study could not demonstrate that self-regulated learning supports higher self-efficacy for helping in cardiac arrest in students. Self-regulated learning is an effective alternative to instructor-led training in BLS and may be feasible to realise for lay-persons like teachers. For male students self-regulated learning seems to be beneficial to support learning and long-term retention of skills. For female students the method of training seems less important.

## Data Availability

Relevant data is included within the body of this manuscript. All raw and analysed data and materials are securely held on a password protected computer system in the Department of Anaesthesiology of the University Hospital Hamburg-Eppendorf (where the study was completed). For further information requests can be made to the corresponding author.

## Abbreviations

AED: Automated External Defibrillator
BLS: Basic Life Support
CPR: Cardiopulmonary resuscitation
OSCE: Objective Structured Clinical Examination
SE: Self-efficacy
